# Triple valve endocarditis by mycobacterium tuberculosis. A case report

**DOI:** 10.1186/1471-2334-12-231

**Published:** 2012-09-27

**Authors:** Quratulain Shaikh, Faisal Mahmood

**Affiliations:** 1 Department of Medicine, Aga Khan University Hospital, Stadium Road, PO BOX: 3500, Karachi, 74800, Pakistan

**Keywords:** Immunocompetent, Endocarditis, Mycobacterium tuberculosis

## Abstract

**Background:**

Granulomas caused by Mycobacterium Tuberculosis have been observed at autopsy in the heart, pre-dominantly in the myocardium and endocardium, but rarely involving the coronary vessels and valvular structures. Mycobacterium tuberculosis valvular endocarditis is extremely rare, with most reports coming from autopsy series.

**Case presentation:**

We report the case of a 17 year old immunocompetent girl who presented with history of fever, malaise, foot gangrene and a left sided hemiparesis. On investigation she was found to have infective endocarditis involving the aortic, mitral and tricuspid valves. She had developed a right middle cerebral artery stroke. She underwent dual valve replacement and tricuspid repair. The vegetations showed granulomatous inflammation but blood cultures and other biological specimen cultures were negative for any organisms. She was started on antituberculous treatment and anticoagulation.

**Conclusion:**

This is the first reported case of triple valve endocarditis by Mycobacterium Tuberculosis in an immunocompetent host. Especially important is the fact that the right heart is involved which has been historically described in the setting of intravenous drug abuse.

This implies that Tuberculosis should be considered in cases of culture negative endocarditis in endemic areas like Pakistan even in immunocompetent hosts.

## Background

Granulomas caused by Mycobacterium Tuberculosis have been observed at autopsy in the heart , pre-dominantly in the myocardium and endocardium, but rarely involving the coronary vessels ,and valvular structures [1]. Mycobacterium tuberculosis valvular endocarditis is extremely rare, with most reports coming from autopsy series [2,3]. Mycobacterium tuberculosis endocarditis has been reported only in the setting of miliary tuberculosis and after aortic valve replacement with infected prosthetic homografts and has been generally diagnosed postmortem [4].

## Case presentation

17 year old girl presented to Emergency department with fever, malaise for 3 months, left foot gangrene for 2 months, left sided hemiparesis for 4 days. A diagnosis of Infective Endocarditis was made on Transthoracic echo and percutaneous embolectomy was attempted at another hospital which was complicated by a left hemiplegia. On examination she was found to be tachycardiac, tachypneic, hypotensive and febrile. GCS was 4/15 and she had a left hemiplegia. Left foot showed gangrene of the medial 3 digits and peripheral pulses including the posterior tibial and dorsalis pedis of the left were weak. There was a pansystolic murmur at the apex with radiation to the axilla and another early diastolic murmur at the aortic area radiating to the carotids. CT scan brain was done that showed a right middle cerebral artery stroke. Transthoracic echo was done which revealed large vegetation on both mitral valve leaflets with a size of 32 X 15 mm on the posterior mitral leaflet ( Additional file 1: Pic. 1). Vegetation was also seen at the septal leaflet of tricuspid valve and aortic valve.

**Figure 1:**
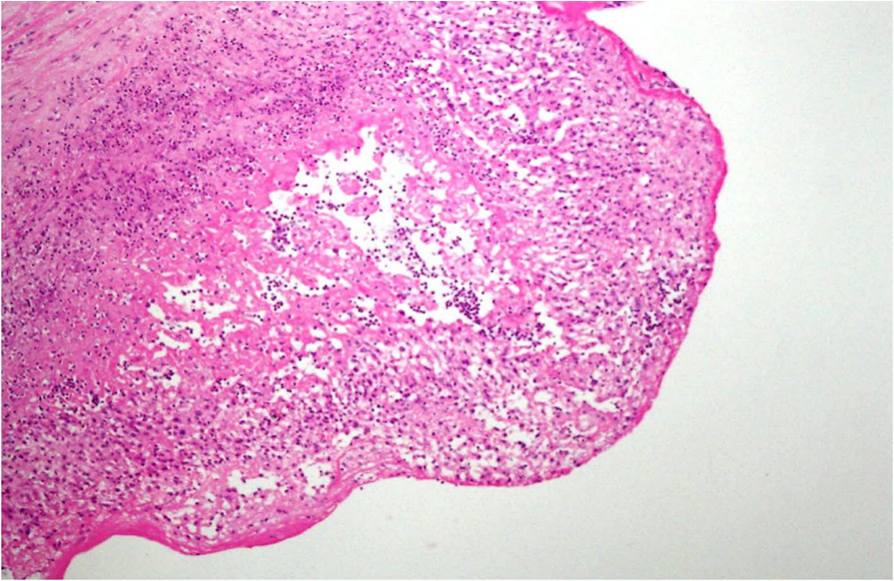
Mitral Valve showing acute and chronic granulomatous inflammation.

**Figure 2:**
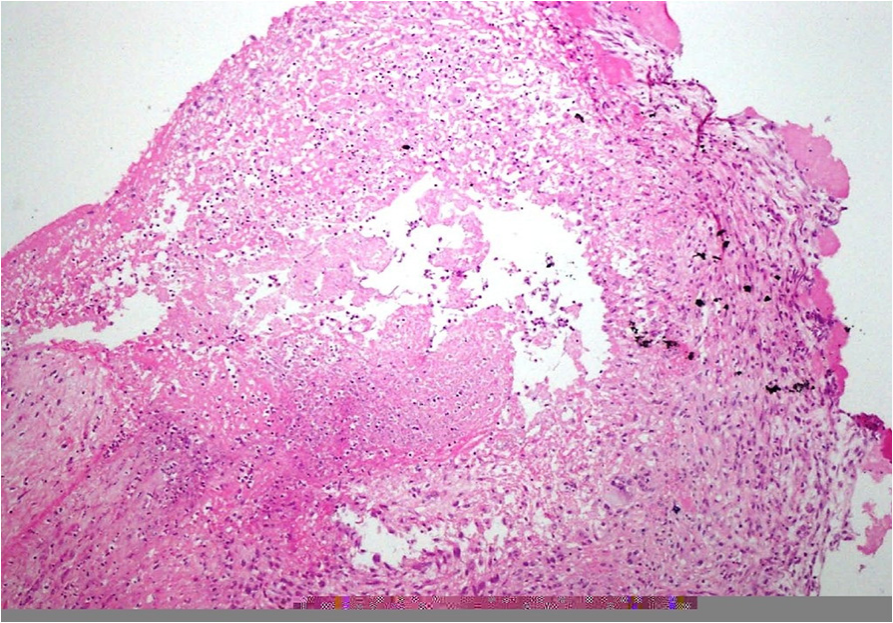
Aortic Valve showing Giant cells and epitheloid cells.

Histopathology of her vegetations revealed moderate acute and chronic inflammation with fibrin and granulation tissue formation along with granulomas. The mitral valve showed focal necrosis with calcification (Figures 1 and 2). The granulomas were composed of typical epitheloid cells and multinucleated giant cells. Cultures of the aortic valve for Acid Fast Bacilli were negative.

She underwent dual valve replacement for mitral and aortic valves and the tricuspid valve was repaired surgically as it was anatomically intact ( Additional file 1: Pics. 2, 3). She was weak on her left side and required regular physiotherapy. Antituberculous therapy was started with anticoagulation.

## Discussion/Conclusion

We report a case of infective endocarditis along with severe aortic regurgitation found to have Tuberculosis on histopathology of the vegetations. To the best of our knowledge this is the first report of Mycobacterium tuberculosis causing valvular endocarditis involving multiple valves in an immunocompetent patient.

Cardiac tuberculosis was recognized and described by Laennec in 1826, assigning the heart as the 13th organ affected in order of frequency [5]. Valvular endocarditis caused by M. Tuberculosis is extremely rare, and is reported only in the context of miliary tuberculosis. Cope et al. reported a case of disseminated tuberculosis with echocardiographically documented aortic valvulitis which resolved on antituberculous therapy [6]. Three cases of right sided tuberculous endocarditis in human immunodeficiency virus (HIV) positive intravenous drug abusers with disseminated tuberculosis have been observed. Endocardial miliary tubercles, polypoidal tubercles that resemble myxoma, nodules on valves, and thrombi containing entrapped tubercle bacilli have been described histopathologically. There are no reports in the literature of valvular endocarditis due to Mycobacterium tuberculosis without miliary tuberculosis in an immunocompetent patient. Another recent case has been reported of an infant with intracardiac tuberculoma in the background of miliary tuberculosis [7]. Valvular endocarditis due to Mycobacterium tuberculosis in immunocompetent hosts should be added to the list of potential manifestations of Mycobacterium tuberculosis infections. Our patient did not manifest any involvement of the myocardium or pericardium although tuberculous pericarditis is a commoner clinical entity in this part of the world, the reasons for which are unclear.

Soyer reported a case of aortic insufficiency treated surgically in which Mycobacterium tuberculosis was recovered from the valve, although no vegetations were present. Another recently reported case from our centre showed culture positive tuberculous endocarditis in a 30 year old immunocompetent host but involved the mitral valve only [8]. There are no cases in literature that report multivalvular involvement including involvement of the right heart in immunocompetent host by tuberculosis in the absence of disseminated disease.

## Consent

Written informed consent was obtained from the patient’s brother for publication of this Case report and accompanying images. A copy of the written consent is available for review by the Editor-in-Chief of this journal. Verbal consent was taken from the patient but as she was not literate, she could not sign the document so a signed consent was taken from her next of kin which was the brother.

## Abbreviations

GCS: Glasgow coma scale; CT: Computed tomography; HIV: Human immunodeficiency virus.

## Competing interests

The authors did not receive any research support for this manuscript. The authors declare that they have no interests interests.

## Authors' information

QS has done her MBBS and FCPS in Internal Medicine. She is currently a fellow in Neurovascular Medicine in Aga Khan University Hospital, Karachi, Pakistan.

FM has done his MBBS followed by MD. He is a Diplomat American Board in Internal Medicine, Infectious Disease. He is currently an Assisstant Professor, Department of Medicine in the Aga Khan University Hospital.

## Authors' contributions

QS did the literature search and drafted the manuscript. FM conceived the case report and provided guidance for drafting the manuscript. All authors read and approved the final manuscript.

## Pre-publication history

The pre-publication history for this paper can be accessed here:

http://www.biomedcentral.com/1471-2334/12/231/prepub

## Supplementary Material

Additional file 1 **Pic.1 Mitral and Aortic Valves showing Vegetations on Transthoracic Echocardiogram.Pic. 2** Patient on cardiopulmonary bypass Mitral Valve exposed via trans septal approach. **Pic.3** Mitral, Aortic and Tricuspid valves exposed during surgery. Click here for file

## References

[B1] Jagirdar J Zagzag D Rom WN, Garay S Pathology and insights into pathogenesis of tuberculosis Tuberculosis 1996 Boston: Little, Brown and Co 467 482

[B2] Suzman S Tuberculous endocarditis Br Heart J 1943 5 19 10.1136/hrt.5.1.19 18609916 PMC503507

[B3] Rumisek JD Albus RA Clarke JS Late Mycobacterium chelonei bioprosthetic valve endocarditis: activation of implanted contaminant? Ann Thorac Surg 1985 39 3 277 279 10.1016/S0003-4975(10)62596-9 3977472

[B4] Kannangara DW Salem FA Thadepalli H Cardiac tuberculosis: tuberculosis of the endocardium Am J Med Sci 1984 287 45 47 10.1097/00000441-198405000-00016 6731481

[B5] Laennec RTH De l’auscultation mediate ou Traite du Diagnostic des Maladies des Poumon et du Coeur 1819 1 Paris: Brosson & Chaudé

[B6] Cope AP Herber M Wilkins EGL Valvular tuberculous endocarditis: a case report and review of literature J infection 1990 21 293 296 10.1016/0163-4453(90)94029-Y 2125624

[B7] Cantinotti M Intracardiac left atrial tuberculoma in an eleven-month-old infant: case report BMC Infect Dis 2011 11 359 10.1186/1471-2334-11-359 22208878 PMC3268750

[B8] Sultan FA Fatimi S Jamil B Moustafa SE Mookadam F Tuberculous endocarditis: valvular and right atrial involvement Eur J Echocardiogr 2010 11 4 E13 10.1093/ejechocard/jep202 20007719

