# Supplementary material for: Triple valve endocarditis by mycobacterium tuberculosis. A case report
Source: BMC Infect Dis. 2012 Sep 27;12:231. doi: 10.1186/1471-2334-12-231 (PMC3511257; doi:10.1186/1471-2334-12-231)
Supplement: Additional file 1 — Pic.1 Mitral and Aortic Valves showing Vegetations on Transthoracic Echocardiogram.Pic. 2 Patient on cardiopulmonary bypass Mitral Valve exposed via trans septal approach. Pic.3 Mitral, Aortic and Tricuspid valves exposed during surgery. [file 1471-2334-12-231-S1.doc]

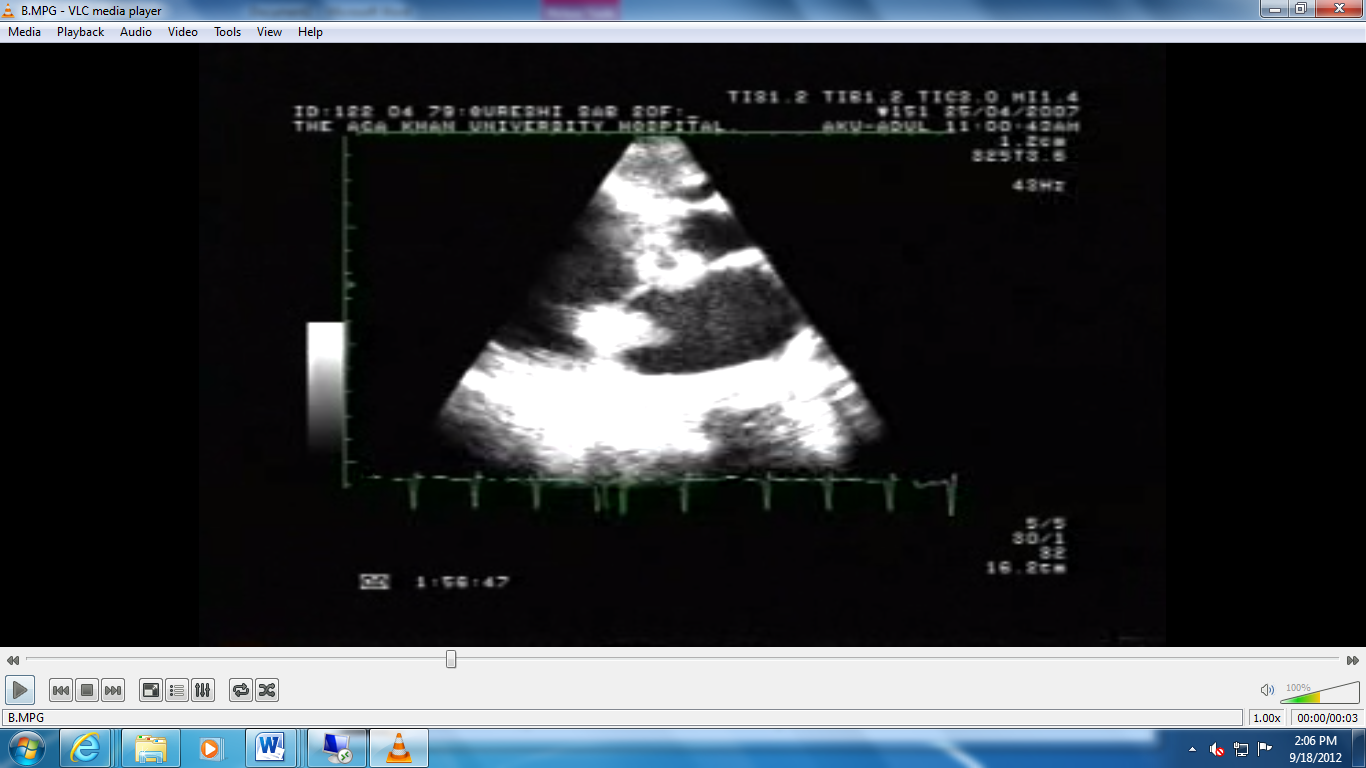


Pic.1 Mitral and Aortic Valves showing Vegetations on Transthoracic Echocardiogram


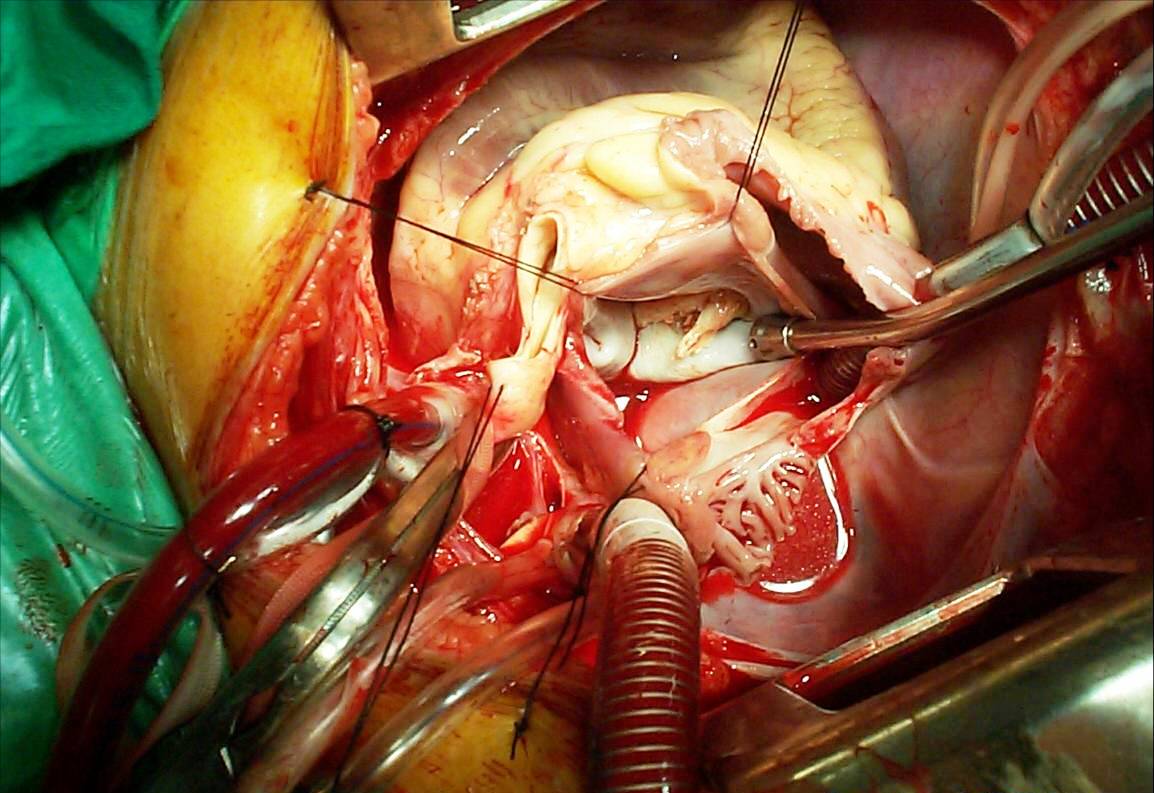


Pic. 2 Patient on cardiopulmonary bypass Mitral Valve exposed via trans septal approach


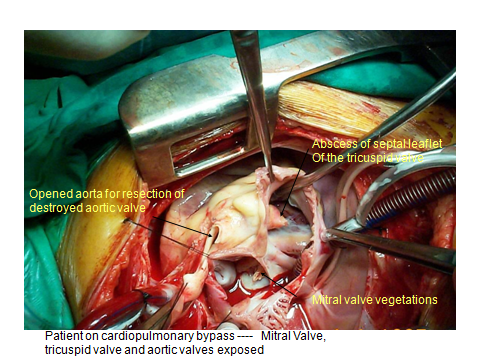


Pic.3 Mitral, Aortic and Tricuspid valves exposed during surgery
